# Management and potentialities of primary cancer cultures in preclinical and translational studies

**DOI:** 10.1186/s12967-017-1328-z

**Published:** 2017-11-07

**Authors:** Giacomo Miserocchi, Laura Mercatali, Chiara Liverani, Alessandro De Vita, Chiara Spadazzi, Federica Pieri, Alberto Bongiovanni, Federica Recine, Dino Amadori, Toni Ibrahim

**Affiliations:** 1 Osteoncology and Rare Tumors Center, Istituto Scientifico Romagnolo per lo Studio e la Cura dei Tumori (IRST) IRCCS, Via Piero Maroncelli 40, 47014 Meldola, FC Italy; 2 Pathology Unit, Morgagni-Pierantoni Hospital, Via Carlo Forlanini 34, 47121 Forlì, Italy

**Keywords:** Primary culture, Cancer microenvironment, Management, Patient-derived xenograft, Zebrafish

## Abstract

The use of patient-derived primary cell cultures in cancer preclinical assays has increased in recent years. The management of resected tumor tissue remains complex and a number of parameters must be respected to obtain complete sample digestion and optimal vitality yield. We provide an overview of the benefits of correct primary cell culture management using different preclinical methodologies, and describe the pros and cons of this model with respect to other kinds of samples. One important advantage is that the heterogeneity of the cell populations composing a primary culture partially reproduces the tumor microenvironment and crosstalk between malignant and healthy cells, neither of which is possible with cell lines. Moreover, the use of patient-derived specimens in innovative preclinical technologies, such as 3D systems or bioreactors, represents an important opportunity to improve the translational value of the results obtained. In vivo models could further our understanding of the crosstalk between tumor and other tissues as they enable us to observe the systemic and biological interactions of a complete organism. Although engineered mice are the most common model used in this setting, the zebrafish (*Danio rerio*) species has recently been recognized as an innovative experimental system. In fact, the transparent body and incomplete immune system of zebrafish embryos are especially useful for evaluating patient-derived tumor tissue interactions in healthy hosts. In conclusion, ex vivo systems represent an important tool for cancer research, but samples require correct manipulation to maximize their translational value.

## Background

**Fig. 1:**
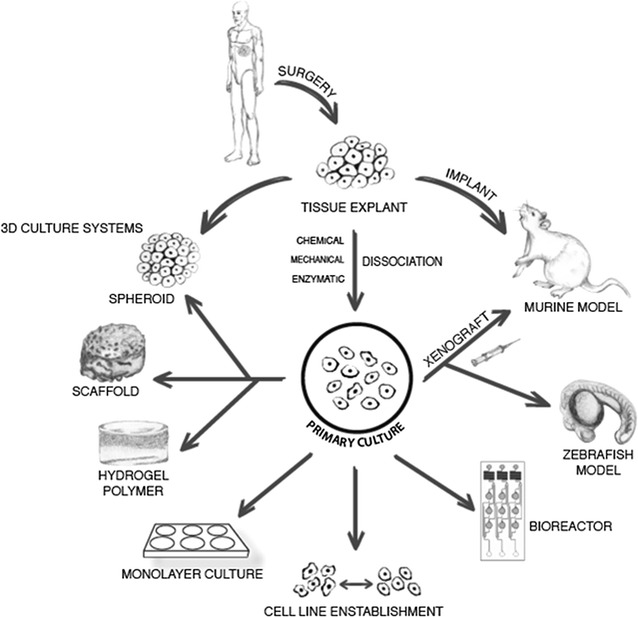
Options for the management of primary cultures

Primary cancer cell cultures are ex vivo cell populations recovered directly from fresh surgically resected tissue samples [1]. These specimens partially reproduce the natural in situ microenvironment of the disease, maintaining the crosstalk between malignant and healthy components [1]. Such features are known to be involved in the different responses to therapies and in all the stages of the natural history of malignant tumors, i.e. cancerogenesis, migration, progression and metastatic dissemination [2–5]. Immortalized cell lines, which represent the most widely used culture method in preclinical assays, are not always predictive of the real cancer behavior [6, 7]. Thus, ex vivo models permit a more faithful reproduction of tumors and are a valid tool for clinical and preclinical analyses [6]. This review defines the main features of primary cancer cell cultures, provides an overview of the different methods for their selection and management, and summarizes the wide range of studies that can be performed with them to improve our understanding of cancer processes (Fig. 1).

## Cancer microenvironment

Cancer is a dynamic disease and represents the second cause of mortality in humans, mainly due to the presence of metastatic disease at diagnosis and the failure of clinical treatments [3]. Tumors are composed of a heterogeneous population of malignant subclones that proliferate and disseminate through crosstalk with other tissues [8]. The development of a cancerous lesion modifies tissue physiology and drives cell phenotype alterations [4] and tumor–stroma interactions that lead to the release of cytokines, chemokines and growth factors [9]. In the absence of pathological conditions, stroma components play a key role in regulating tissue homeostasis and maintaining the endothelial structure integrity [2].

The physical properties and functions of the ECM serve as an anchor point for the cells and as direct modulator of cellular behavior. This cell–matrix crosstalk regulates pathways involved in cell proliferation, differentiation and migration, creating a favorable microenvironment for cancer progression and metastatic propagation [10, 11]. The ECM is also a reservoir for growth factors that are released upon tissue request [12]. Among these, the interleukin family and transforming growth factor-beta (TGF-β) are known to be involved in the majority of carcinogenesis processes including the regulation of immune cell functions [4, 13–15], neo-angiogenesis [16] and the manipulation of the tissue scaffold structure [15]. Moreover, cancer cells overexpress numerous factors capable of modifying the ECM architecture. Matrix metalloproteinases (MMPs) and LOX family oxidases are two groups of enzymes that alter the mechanical properties of the tumor niche [4]. The LOX family regulates the structural integrity and tensile strength through the crosslinking and stabilization of collagen. The hypoxic condition characterizing the cancer microenvironment, induces LOX overexpression, enhancing tissue stiffness and ECM rigidity [17]. These altered mechanical forces have been shown to increase cancer cell proliferation and to stimulate migration to the surrounding tissues [18].

Among stroma components, fibroblasts play a central role in tumor growth, motility, angiogenesis, and metastatic dissemination [2, 19–21]. They may acquire a malignant phenotype through the shift to cancer-associated fibroblasts (CAFs). This altered subpopulation contributes to cancer progression [22], with paracrine secretion of pro-cancer factors (e.g. HGF, TGFβ, VEGF, NK4) [23], deposition and remodeling of the ECM [22] and immune regulation [24]. CAFs, like tumor cells, also release MMPs. As previously mentioned, MMPs are involved in the mechanical alteration of tissue scaffolds but also play a role in other processes such as immune regulation, angiogenesis, intravasation, pre-metastatic niche induction and inflammatory regulation [25–27]. CAF-mediated immune regulation modulates both innate and adaptive immunity. These processes are stimulated by the secretion of chemokines that recruit cell populations (e.g. monocytes and neutrophils) and activate their immunosuppressive phenotypes [27, 28]. Moreover, malignant cells promote the activation of the NF-κβ pathway, resulting in pro-inflammatory signaling. This leads to the dysfunction of immune cell regulation, including B cell growth and CD4 T-cell differentiation [29]. CAFs have also been shown to have a heterogeneous origin that depends on the site of onset of the cancer [24, 30]. CAF transdifferentiation can occur in several cell types. The main sources of CAFs are normal fibroblasts and mesenchymal stem cells (MSCs), but stellate cells, fibrocytes, endothelial and epithelial cells can also differentiate into activated fibroblasts [30–33].

Developed over a decade ago, the cancer stem cell (CSC) theory posits that the tumor has its own stem cells with self-renewal ability and ascribes the origin of these malignant cells to alterations of regulation pathways through genetic mutations of the healthy stem cell population [34]. CSCs represent the crest of a hierarchy that defines them as the initiating progenitors of cancer and as the cells responsible for the generation of different cancer subclones. The microenvironment of the tumor niche is essential for the maintenance of CSC stemness. In this context, CAFs activate pathways involved in the stimulation of stemness properties, including the NOTCH and WNT signaling cascades. The NOTCH pathway contributes to the inhibition of cell differentiation, whereas WNT promotes the development of CSCs originating from normal stem cells and non-stem cancer cells [35, 36]. Hypoxia also contributes to the dedifferentiation of cancer cells in CSCs [37], the decrease in oxygen stimulating epithelial mesenchymal transition (EMT), a process involved in metastatic development. EMT regulates cancer cell plasticity, inducing CSC phenotype differentiation and self-renewing properties in non-stem cancer cells [38, 39]. There is ample evidence of the role of CSCs in resistance to chemotherapy, both in hematologic and solid malignancies [40–43]. The low drug sensitivity is attributed to a higher EMT-associated gene expression, enhanced synthesis of drug efflux-related transmembrane protein transporters (ATP-binding cassette) and antiapoptotic proteins [40, 44, 45].

The cancer crosstalk with healthy components includes many other cell populations, e.g. MSCs, adipocytes, endothelial progenitor cells (EPCs), macrophages and myeloid-derived suppressor cells (MDSCs), all of which are involved in various stages of the disease [3, 46–49]. In conclusion, the substantial contribution of normal tissue components in tumor processes is evident and must be taken into consideration in in vitro and in vivo experimentation.

## Reproduction of tumor microenvironment by ex vivo cultures

Up to now, in vitro and in vivo cancer research has largely depended on the use of immortalized cell lines, which are usually cultured on monolayer supports for in vitro assays or xenografted onto immunocompromised animals for in vivo evaluations [50–53]. Cell line models have the advantage of guaranteeing a large amount of material (depending on their immortalized nature) which permits amplification and sample storage and enables a large number of experiment replications to be performed. Moreover, these models are simple to use and manage, ensuring a prolonged conservation of molecular and genetic features. Such characteristics make cell lines a strong and reliable system for biologic evaluations in the laboratory where they remain the most practical and least expensive tool for preclinical studies [54]. Furthermore, cancer cell lines are usually commercially available, thus representing a valid means of confirming and reproducing results obtained in laboratories all over the world.

However, cancer cell lines also have some limitations. The homogeneity of cell line populations and the high number of passages in monolayer systems make these models a far cry from the actual disease. Cell line cultures do not faithfully reproduce the molecular crosstalk, cell–cell interactions and tissue morphology that characterize the tumor microenvironment [7, 55], resulting in major gaps in our understanding of the pathway modulations believed to play a central role in cancer development and propagation [4].

Increasing interest has recently been shown in the ex vivo culture of patient-derived tumor samples [6, 53, 56, 57]. The excision of the malignant and healthy tissue guarantees the preservation of cell phenotypes and the heterogeneity of cancer subpopulations, both of which help to mimic the tumor microenvironment. Primary cultures preserve cancer cells with stem-like phenotypes, an advantage not always offered by cell lines. This acquires particular relevance in preclinical research because CSCs are known to play an important role in mechanisms of drug resistance [40]. Different research groups have also reported the presence of CSCs in cell lines, demonstrating the conservation of self renewal properties over time on in vitro devices [58, 59]. However, not all cell lines have CSCs, including those of Wilms’ tumor, rhabdomyosarcoma and osteosarcoma [59]. For this reason, and for the long periods of in vitro culture, cell lines are not ideal sources of CSCs.

Moreover, the availability of fresh samples enables single tumors to be studied and facilitates comparisons between lesions in the same or different parts of the body [60]. For example, Daigeler et al. compared gene expression in 19 liposarcoma primary cultures (12 primary tumors, 6 recurrences and one metastasis) after treatment with doxorubicin [61]. The results showed that these primary cultures preserved the same degree of response observed in clinical practice for the different stages of disease, making them an efficient tool for the in vitro reproduction of the effect of drugs on patients [61]. Thus, ex vivo systems not only represent an important preclinical tool to evaluate the response of human cancer cells to drugs, but also a promising methodology to improve personalized treatments [62].

## Management of primary cultures

The use of primary cancer cultures in preclinical studies, which requires the approval of the local Ethics Committee, is dependent on the availability of surgical material [54]. A key figure in this type of preclinical research is the pathologist because of his/her expertise in sample selection and histomolecular classification, especially for tumors that do not have specific markers [63]. The establishment of a primary culture starts with the manipulation of fresh tissue samples. In this review, we describe a number of protocols for the management of ex vivo material that can be used in in vitro and in vivo preclinical studies (Fig. 1).

The first step after surgical dissection consists in tissue dissociation to obtain a single cell suspension [64]. The three main dissociation techniques are based on chemical, mechanical and enzymatic processes. Chemical dissociation involves the sequestration of cations implicated in the conservation of the intracellular 3D structure and is achieved by exposing the tissue to reagents (e.g. EDTA or EGTA), contributing to the degradation of intercellular connections [65]. Mechanical dissociation involves the homogenization of the solid tumor sample by manual mincing. This is achieved by disaggregation of the tissue into small fragments using a sterile scalpel or scissors followed by filtration and vortexing passages to obtain a final single cell suspension [66]. Salawu et al. established seven soft tissue sarcoma cell lines by manually mincing the ex vivo material into small pieces, centrifuging once and seeding the final supernatant in flasks [67]. One of the disadvantages of mechanical manipulation is the release of degrading enzymes produced by the traumatic incisions that damage cell components and contribute to a low percentage of cell survival [68]. Enzymatic dissociation is an alternative method for the digestion of biological tissues and takes advantage of the action of enzymes with different targets [68]. Both mechanical and enzymatic digestion of the surgical material is often used before seeding. After cutting the specimen into small fragments, enzymatic disaggregation is carried out using specific selection of enzymes, concentrations and exposure times to obtain the best cell yield [69]. There is still no a standardized protocol for this. For example, Theerakitthanakul et al. digested fresh specimens of Wilms’ tumor for 3 h at 37 °C in 160 μg/mL of collagenase A [70], whereas Wang et al. processed primary breast tumors for 40–60 min at 37 °C in a 1:1 solution of collagenase/hyaluronidase [71]. In a recent work we incubated minced samples of breast cancer-derived bone metastasis for 2 h with 2 mg/mL of collagenase type I (1:1 in D-MEM H) at 37 °C [72].

The number of cells obtained depends on the amount and condition of surgical tissue available. Faili et al. compared different digestion approaches to analyze the viability rates of 20 tissue samples of resected colorectal cancer [60]. They reported that enzymatic dissociation with trypsin on an agitator at 37 °C for 100 min produced 68–74% of viable cells, whereas mechanical disaggregation, collagenase type H (1.5 mg/mL) and trypsin on shaking water (37 °C) obtained a lower yield [60].

The composition of primary cultures varies, mainly on the basis of the tissue of origin. Different cell populations do not have the same ability to grow for long periods on in vitro devices or are not capable of attaching onto synthetic surfaces. Hematopoietic and stromal cells are often present after sample digestion [73]. For example, fibroblasts adapt extremely well to the in vitro environment and, as explained in the next paragraph, their rapid overgrowth may be problematic for the preservation of cancer cells. The malignant components of primary cultures reflect the heterogeneity of subclones composing the tumor mass and include a variable portion of CSCs. Solid tumors express different stem-specific markers, depending on the site of origin [74]. There are several techniques for identifying malignant stem subclones. Fluorescence-activated cell sorting (FACS) and magnetic cell sorting are two methodologies that take advantage of stem cell-related antigens through antibody selection [74].

Another critical area for the correct management of primary cultures is the preservation of the tumor microenvironment following the use of early passages for downstream analyses. A small number of passages does not generally cause phenotype alteration in cell lines but have a drastic impact on the nature of the primary culture. In fact, early cell passaging leads to changes in gene expression, proliferation rate and drug response that enrich some subclones but not others, altering the high translational value of this model. Thus, the first steps of primary culture manipulation are of prime importance if useful translational results are to be achieved [75].

As described above, the molecular crosstalk with non malignant cells and the presence of stem-like subclones are important aspects that reflect the nature of the tumor. Although the heterogeneity of cell populations contributes to mimicking the tumor microenvironment, elements such as nutrient perfusion or 3D structures cannot be reproduced by ex vivo samples. Some of these disadvantages can be overcome by using innovative in vitro and in vivo systems, described below. Indeed, new approaches are constantly being developed to improve the potentialities of primary cultures in different areas of preclinical research. However, the choice of the model must be correlated with the aim of the study to maximize the advantages of using cells directly recovered from patients.

## Establishment of immortalized cancer cell lines

We have already seen the advantages and disadvantages of working with cell lines. Whilst they are undoubtedly an important tool for cancer research, their limitations must be borne in mind when analyzing results. Furthermore, commercial cell lines are not available for all stages of disease or for all tumor types [76], making it essential to establish and characterize new cell lines from fresh human or animal tissue [67, 77]. The process that induces primary culture cancer cells to grow and acquire an immortalized phenotype is complex and results are unpredictable [78]. The capacity to grow on plastic supports varies on the basis of the cancer cell histotypes and depends on lesion aggressiveness. In agreement with some authors, we observed (data not shown) low mitotic activity in neuroendocrine tumor (NETs) cells which was reflected in a low proliferation index on 2D devices [79]. This low growth rate may have been due to an intrinsic phenotypic and genetic aberration or to inappropriate nutritional support [80].

The establishment of an immortalized cancer cell line includes three key events: (1) selection of cancer cells from stromal cells; (2) cell immortalization; and (3) morphological and biomolecular characterization of the cell line. The tumor fraction must also be separated from the various components of the original tissue. The rapid proliferation of fibroblasts favored by cancer cell-secreted growth factors is one of the main problems of this process [81, 82]. Geneticin is one of the options used to eliminate fibroblasts. This antibiotic has a selective action on fibroblasts and contributes to controlling their overgrowth, with little interference on cancer cell survival [83]. As fibroblasts are more sensitive to trypsin than to cancer cells, another option is to administer trypsin sequentially and recover the detached cells [84].

Immunomagnetic separation is another method used to isolate cancer cells from fresh tissue, peripheral blood or bone marrow [85]. Saalbach et al. developed the fibroblast-specific monoclonal antibody (MAb) AS02 immobilized on goat-anti-mouse-magnetic beads which is capable of purifying cell cultures from fibroblast contamination [86]. A “negative sorting” approach can also be used if antibodies are not available for specific cancer subtypes. This method removes non-tumor components by the addition of different beads covered with antibodies directed against healthy cells [87, 88]. Schreier et al. characterized anti-CD45 magnetic beads for the selection of CD45-positive cell types, e.g. fibroblasts or leukocytes [89]. A critical issue in immunomagnetic sorting is the breaking of antibody–antigen bonds at the end of the sorting process because of the impact of high bead density on culture cell proliferation. The degree of purity of the suspension depends on the amount of cell clusters and unwashed cells attached to the surface of the beads [90].

Different approaches have been validated to develop immortalized phenotypes [91]. Infection with a viral vector can be used to transfect oncogenes that play a part in deregulating the pathways involved in cell cycle control. The transfection of telomerase or telomerase reverse transcriptase (TERT), involved in the elongation of telomeres and in chromosome stability, can be used [91–94]. Immortalization can also occur spontaneously, without genetic alterations [67]. For example, Wei et al. established an ovarian cancer cell line maintaining the population in culture for > 50 passages during more than 2 years [95]. Qin et al. reported establishing a highly metastatic buccal squamous cell carcinoma cell line after 30 passages [96].

The immmortalization of primary cells can be also performed using in vivo models, usually of mice. The microenvironment of live tissue promotes cancer cell growth mainly because of the high number of epithelial components and the consecutive crosstalk with the tumor [97]. Human cancer cells are grafted as small fragments or as single-cell suspensions onto the animal’s body and the relatively large size of mice makes it possible to remove the tumor mass and repeatedly engraft the digested resection to obtain the stabilization of the cell line [97]. Cavalloni et al. used NOD/SCID mice for a patient-derived intrahepatic cholangiocarcinoma xenograft and re-implanted the primary culture four times before obtaining cell line stabilization [98].

The last step in this process is the morphological characterization of the new line which is needed to validate the cancer phenotype and to exclude cross-contamination by other cell components [67]. This is problematic for cancer histotypes that are not recognizable by a specific marker and requires the assistance of an expert pathologist to discriminate between benign and malignant cells. Molecular characterization using flow cytometry is essential to evaluate the genetic features of the cancer cells, as is immunochemistry or PCR to determinate the expression of biomarkers [67, 99].

## 3D primary culture models

Tumor lesions are dynamic masses of malignant and healthy cell populations in continuous co-evolution and supported by an extracellular 3D matrix that is constantly being remodeled. These alterations in the tissue structure are driven by the nature of the disease which modifies the matrix scaffold [100]. Thus, the complexity of the stroma and its interaction with disease components makes it virtually impossible for monolayer models to faithfully reproduce the real tumor microenvironment [100]. To overcome these problems, a number of 3D models have been developed to more realistically mimic the tumor niche structures. The combination of 3D technologies and primary cultures represents one of in vitro models that comes closest to reproducing the real pathophysiological features of the tumor.

Synthetic matrices have been extensively studied as tools for the reproduction of cancer architecture, and polymer hydrogel systems are one of the resulting technologies. These engineered gels possess some of the properties of the tumor niche, e.g. stiffness. One option for synthesis is the use of end-functionalized multiarm polyethylene glycol (PEG) macromers to generate an inert and hydrophilic platform through the chemical crosslinking of functionalized polymers [101]. Jiglare et al. tested the chemo-radiotherapy sensitivity of 7 primary glioblastoma cultures seeded on hyaluronic acid-rich hydrogel and compared results with those obtained on 2D monolayer supports [102]. The culture on 3D matrix showed a lower response to treatments and the tumor growth rate was comparable to that of clinical data [102].

Biomimetic porous scaffolds belong to the technologies used in tissue engineering applications. The nature of the matrix composition can be selected from a pattern of synthetic polymers, i.e. poly(d,l-lactide) (PDLLA), poly(l-lactic-co-glycolic acid) PLGA, polystyrene (PS), poly(methyl-methacrylate) (PMMA), poly(caprolactone) (PCL) and polyurethane (PU), non-polymeric materials (collagen, fibronectin, Matrigel or hydroxyapatite), or substances derived from biological samples [103]. The biologically derived matrix materials provide a useful platform for the reproduction of the interactions between cancer cells and the extracellular matrix (ECM). Collagen-based scaffolds are widely used as an ECM mimetic device as collagens are the most abundant proteins in connective tissues [104]. We previously developed a 3D collagen-based culture system of primary liposarcoma and compared it with patient histology [105]. Morphological and genetic features were maintained in the 3D culture, and the MDM2 liposarcoma marker was highly overexpressed, suggesting collagen scaffolds are a promising tool to mimic cancer tissue [105]. Moreover, scaffold devices are suitable models for stem cell enrichment, improving EMT and CSC properties [106, 107]. In this context, scaffolds provide useful platforms to study radiation and drug resistant processes driven by CSCs. 3D scaffolds have been shown to mediate the enrichment of cancer cells and CSC subclones with respect to the other components of primary cultures, increasing the amount of tumor material obtained by sample digestion [105]. In addition to the matrix reproduction, this enrichment makes 3D scaffold cultures a useful starting point for preclinical analysis.

Spherical cancer models made their appearance four decades ago and are still used today [108]. As there is still not an official nomenclature to distinguish between the different types or biological origins of these systems (e.g. *organoid* or *tumorsphere*), we use the term ‘spheroids’ to refer to all spherical cancer models. These 3D cultures originate from cell–cell interactions that drive the aggregation processes of clusters or single cells in spherical structures. The spherical nature of these systems leads to the development of inner and superficial zones with different phenotypic and biological features [109]. For example, the exponential phase of proliferating cells is slowly replaced by stable growth, leading to an increase in the proportion of quiescent cells that closely mimics disease progression in humans [110]. Primary cultures are one of the biological starting materials used for the generation of spheroids. Halfter et al. compared the chemosensitivity of spheroids derived from HER2- positive breast cancer cell lines with that of spheroids derived from 120 fresh tissue samples [111]. Their results highlighted a greater efficacy and lower metabolic activity of the spheroids derived from primary cultures than those originating from cell lines [111]. Qureshy-Baig et al. reported that primary colorectal cancer spheroids maintained their chemoresistance and genetic mutations with respect to the tissue of origin [112].

Different protocols have been validated for the generation of spheroids on the basis of the tumor site [113]. For primary samples, the main difference in the processing methods used lies in the degree of tissue dissociation. We previously described how to obtain a single cell suspension via the digestion of minced tumor samples. Following this step, a spheroid is generated by cultivating the cells at low cell density and in low-adherent conditions to allow cells to float and avoid aggregation into clusters [113]. The aim of this method is to obtain spheres derived from the clonal expansion of a single cell and, consequently, to have a number of 3D tumor subclonal populations available for use. For this reason, culture medium is not supplemented with fetal bovine serum (FBS) but rather with growth factors that support stem cell proliferation, e.g. epidermal growth factor (EGF) and fibroblast growth factors (FGFs) or hormones such as insulin, progesterone and hydrocortisone [114]. Moreover, culture surface, medium composition, time of development and cell density are all taken into consideration to enhance spheroid generation [113]. This protocol can be used for the selection of cancer cells with stem-like properties. Indeed, only stem/progenitor cells are capable of developing into spheroids in serum-free media [115]. This system represents an optimal tool to expand and isolate stem-like subclones from other malignant subclones.

Unlike cell lines, ex vivo material cannot be totally dissociated into single cells. The process of finely cutting and crushing the culture in flasks containing medium supplemented with FBS is sufficient to guarantee the compaction and remodeling of cells into spheres [116]. A strainer with pore sizes ranging from 40 to 100 µm facilitates the selection of aggregates that can be maintained in non-adherent conditions. For example, Morales et al. left inflammatory breast cancer spheroids seeded in 1% agarose-coated tissue culture plates in culture for up to 3 months [117].

Another method used to generate spheroids is the direct culture of tissue fragments in which the tumor tissue is cut into pieces of roughly 0.3–0.8 mm^3^ in size which are then seeded in culture flasks coated with 0.75% agar plunged in medium supplemented with an excess of nonessential amino acids [118]. Depending on the nature of the tissue, the fragments acquire a spherical shape after a varying number of days and can be selected by eliminating the useless debris. Heimdal et al. generated spheroids from tissue samples of head and neck squamous cell carcinoma with a > 90% success rate [119]. Fragments rounded to a spherical shape within 1 week of culture and after a further 2 weeks reached the final spheroid state [119].

Finally, the main contribution of all of these systems is that they permit the interaction between cancer cells and 3D structures. Some techniques enable all the cell populations of patient tissue to be maintained, e.g. spheroids originating from directly by undigested fragments. Other systems show a preferential selection of malignant components, such as collagen-based scaffolds. Overall, 3D cultures enable us to study the stem-like properties of cancer cells. Sphere-forming assays and CSC enrichment offer a suitable platform to study the impact of cancer stem-like components on resistance to clinical treatments, one of the most important aims of preclinical research.

## Bioreactors as a tool for ex vivo research

The recapitulation of tumor-stroma crosstalk using primary tumor cell populations and a 3D tissue structure lacks a series of biochemical and biophysical stimulations that influence biological processes. Some of these missing parameters can be added and monitored using bioreactor systems, which are devices designed to influence the physiological properties of a cell culture through controlled fluid perfusion [120]. This model permits the regulation of oxygen intake, pH and temperature, simulating a circulatory environment that is controlled by computer hardware. Moreover, the stress and pressure induced by the flow of media influence cellular behavior and alter processes such as migration, cell cycle and proliferation [121]. These dynamic systems have already proven capable of enhancing the survival of cell cultures with respect to common static approaches. Indeed, the constant oxygenation, nutrient support and continuous metabolite clearance produced by the perfusion system simulate a real tissue microenvironment [120]. Moreover, the use of bioreactors facilitates cell culture for long periods (more than 10 days), which explains why bioreactors are widely used in biopharmaceutical industrial processes and tissue engineering applications [122].

As previously mentioned, cell-based assays generally require a large number of cells that are difficult to obtain from limited patient-derived samples. [123]. Two standard bioreactor technologies are stirred culture vessels and rotary systems. The former is a simple device that reproduces a hydrodynamic environment via a controlled stirred tank and spinner vessels, while the latter consists in rotating cylindrical culture containers without internal mechanical inputs [120]. Initially used for microgravity tissue regeneration tests, rotary cell culture systems promote cell–cell aggregations through low turbulence that reproduces a condition of microgravity [124], a requisite for the development of 3D cultures, in particular spheroids [125]. Microbioreactors are the most complex devices in the field of microfluid perfusion, permitting an accurate control of the culture microenvironment and allowing the manipulation of physical and biological parameters. Furthermore, the paracrine and autocrine factors secreted are not diluted and contribute to a more realistic crosstalk between cell populations [126]. The amount and speed of media delivery can be carefully controlled to simulate tissue perfusion of nutrients, proteins or biological factors and flow-induced mechanical strain and shear stress [127]. The latest microfabrication techniques are based on monolayer surface chambers designed to reproduce organ-specific microenvironments [128]. These devices, called “organ-on-chips”, create a complex engineered physiological microenvironment that maintains the versatility of in vitro applications, including the possibility of developing 3D scaffold cultures and spheroids [129, 130]. Ruppen et al. used this technology to test spheroid aggregation in primary cultures of adenocarcinoma and squamous cell carcinoma of the lung using tumor cells alone or tumor/pericyte co-cultures [130]. Although no studies on the use of these organ-on-chips devices in primary cancer cultures have been performed to date, the promising results obtained on cell lines will probably orient the interest of the scientific community in that direction.

In conclusion, bioreactors reproduce tumor microenvironment features that are impossible to recreate using common in vitro methods. Microfluid systems haven’t still highlighted the contribution of non-malignant components of primary samples. On the other hand, the possibility to prolong the time on culture and the dynamic perfusion permit to overcome some of the limitation of ex vivo static methods.

## In vivo models

The use of animal models has long been considered a fundamental tool to better understand the many aspects of human diseases, including cancer. In vivo models provide more realistic data than those obtained from in vitro experiments in that, as complete organisms they reproduce many of the tissues and molecular interactions with malignant cells, resulting in a more faithful reproduction of the human scenario [131]. In this context, primary cancer cells are injected or implanted directly into tissues. Therefore, the site of injection reproduces the microenvironment in which cancer cells interact with healthy tissues and then offer the possibility to monitor processes, such as neoangiogenesis, which are problematic to evaluate in in vitro systems. Several animal species are available for clinical research and this review focuses on two models: mice and zebrafish.

## Mice models in primary culture research

A major contribution to experimental cancer research has been made by genetically engineered mouse models (GEMMs). The anatomy, genetics and physiology of the mouse and human species are well conserved because of the common mammalian origin [132]. The use of PDX techniques in murine engineered systems represents one of the more advanced investigation tools for cancer research as it supports the areas of therapeutics, personalized medicine, drug screening, biomarker development and co-clinical trials [133]. GEMMs have become the main recipients for PDXs and are extensively used for translational investigations by the pharmaceutical industry and in academic research [134–136].

Surgical resection tissue and biopsies are the most widely used materials for PDXs but the injection of cells obtained from drainage fluid is also an option. Mice must be immunocompromised before being injected with human cells. There are several strains of immunodeficient mice such as NUDE (nu), SCID (scid), NOD-SCID and NOD/SCID/IL2λ-receptor null (NSG) which differ on the basis of the lack of one or multiple functional immune components, e.g. T cells, B cells or NK cells [137, 138]. The level of immunosuppression modifies the engraftment rate, and models such as NOD-SCID mice, with impaired NK cells and nonfunctional T and B cells, are particularly appropriate hosts for PDX manipulations. The site of cell inoculation is based on the study aim and is designed to obtain the growth of a tumor mass or a metastatic lesion. When possible, the generation of primary cancers is obtained by orthotopic implantation into the same organ of the murine model as that of the origin of the human lesion, e.g. brain, colorectum, oral cavity, pancreas, thus conferring a translational advantage [139–143]. Conversely, the development of bone or lung metastases can be obtained by intracardiac, intratibial or tail vein injections [144, 145]. Subcutaneous implantation in the dorsal region of the animals is the most common heterotopic graft site [146–148]. Moreover, co-injections of nutrients, such as Matrigel^®^, or subcutaneous implantations of 17β-estradiol (E2) pellets in experiments with ER-positive cell populations, support cell growth and increase the engraftment rate [149–151].

The PDX approach is particularly useful in tumors for which there are no tests available for the detection of markers of sensitivity or resistance to chemotherapy. In a similar scenario, treatment efficacy in mice injected with PDX cells provides important clinical information that facilitates the choice of therapy. Dong et al. performed renal capsule transplantations of non-small cell lung cancers (NSCLC) from untreated patients [152]. The rapid assessment of treatment efficacy (6–8 weeks) and correlation between chemosensitivity and clinical outcome confirmed the predictive efficacy of this model [152].

Such results can also be used to optimize the design of clinical trials. For example, PDXs in GEMM hosts help to identify the subsets of patients who are more sensitive to new drugs and combinations. Furthermore, biologic and genetic analyses of responsive and resistant mice subpopulations facilitate the discovery of new biomarkers associated with therapeutic outcome. Berlotti et al. assessed the response to cetuximab, an anti-EGFR antibody, in 85 metastatic colorectal cancer PDXs and also evaluated genotypic differences [153]. HER2 amplification was observed in the subset of mice that showed resistance to cetuximab, indicating the need for alternative treatment in patients with overexpression of the ERBB2 gene [153].

Murine models also have limitations related mainly to the nature of the implant and to the characteristics of the host chosen as PDX recipient. As previously mentioned, some tumors have a low engraftment rate, i.e. hormone receptor-positive tumors, which puts them at a distinct disadvantage because of the small quantity of material often obtained from primary culture. Moreover, murine stroma in the implant site progressively substitute the human components, altering the tumor phenotype. Some of these changes lead to modifications in paracrine regulation and in the natural crosstalk between the disease and the niche microenvironment, impoverishing the translational value of the model. Finally, the length of time required for the development of tumor masses and the high cost of supporting animal facilities makes this model unfeasible for the majority of preclinical studies.

## Zebrafish xenografts

In the 1960s, Georges Streisenger was the first to describe the potential of a small tropical fish (*Danio rerio)* from Bangladesh and North East India [154] as a new system for studying human diseases, including cancer. The fish, commonly known as zebrafish, has distinct biological advantages as a research model thanks to their evolutionary conservation of the majority of human genetic pathways [155]. Indeed, the sequencing of the zebrafish genome revealed the presence of about 82% of the homologous functional genes involved in human diseases [156]. Moreover, the easy and rapid genetic manipulation of this species, the low cost of husbandry and the availability of several transgenic lines have contributed to its widespread use for preclinical evaluations.

Zebrafish have specific features that make them ideal candidates for PDXs. First, the embryo’s immune system takes a month to fully develop, and this immunosuppressive state is key to preventing the rejection of human tissue engraftment [157]. The transparency of the embryo body is another important characteristic that can be chemically controlled, even after the natural appearance of pigmentation. In this way, body areas are clearly visible, simplifying microinjection. Lee et al. performed the first xenotransplantation of melanoma cancer cells into zebrafish at the blastula stage of development, around 3.5 h post fertilization (hpf) [158]. Their pioneering study provided important evidence of the migration behavior of tumor cells up to 8 days post injection (dpi) and revealed that healthy cells, fibroblasts and melanocytes microinjected into the zebrafish did not show the same migration rates [158]. This approach was subsequently also used for PDXs (Table 1).

**Table 1:** Summary of the methods used for patient-derived xenografts of primary tumor cell cultures in zebrafish

Tumor origin	Origin of species	Sample	Zebrafish line	No. of cells	Stage	Site of injection	References
Abdominal liposarcoma	Human	SR	Tg(Kdrl:mCherry)	50–400	Embryo (2 dpf)	Heart cavity	[105]
Acinar cell carcinomas	Zebrafish	SR	tg(CB1)	2/3 × 10^5^ and fragments	Larvae (7–14 dpf)/adult	Intraperitoneal/abdomen and dorsal muscles	[159]
Acute lymphoblastic leukemia	Zebrafish	SR	AB and EK	5 × 10^5^	Adult	Intraperitoneum	[160]
Acute myeloid leukemia	Human	BM	AB	100–200	Blastula (3 hpf)	Yolk sac	[161]
Ampulla of Vater adenocarcinoma	Human	SR	Tg(fli1:eGFP)/alb(albinos)		Embryo (2 dpf)	Yolk sac	[162]
Ampulla of Vater adenocarcinoma	Human	SR	Tg(fli1:eGFP)	Fragments	Embryo (2 dpf)	Yolk sac	[163]
Bone metastasis	Human	SR	Tg(fli1:eGFP)	50–400	Embryo (2 dpf)	Duct of Cuvier	[72]
Colon adenocarcinoma	Human	SR	Tg(fli1:eGFP)/alb(albinos)		Embryo (2 dpf)	Yolk sac	[162]
Ependymoma	Mouse	SR	Tg(fli1:eGFP)	2 × 10^5^	Juvenile (30 dpf)	Cerebral hemisphere	[164]
Glioblastoma	Human	SR	AB		Embryo (2 dpf)/adult	Brain ventricle	[165]
Glioblastoma	Mouse	SR	Tg(fli1:eGFP)	2 × 10^5^	Juvenile (30 dpf)	Cerebral hemisphere	[164]
Liver metastasis from NET	Human	SR	Tg(fli1:eGFP)	100	Embryo (2 dpf)	Perivitelline space	[166]
Melanoma	Zebrafish	SR	Casper	2 × 10^5^	Adult	Peritoneal cavity/intracardiac cavity	[167]
Multiple myeloma	Human	Pl	Casper	100	Embryo (2 dpf)	Perivitelline space	[168]
Myeloid leukemia	Human	PB	AB	50–200	Embryo (2 dpf)	Posterior cardinal vein	[169]
Pancreatic cancer	Human	SR	Tg(fli1:eGFP)/alb(albinos)		Embryo (2 dpf)	Yolk sac	[162]
Pancreatic cancer	Human	SR	Tg(fli1:eGFP)	Fragments	Embryo (2 dpf)	Yolk sac	[163]
Papillary thyroid cancer	Human	SR	Tg(fli1a:EGFP)^y1^	100	Embryo (2 dpf)	Perivitelline space	[170]
Pituitary adenoma	Human	SR	Tg(fli1:eGFP)	100	Embryo (2 dpf)	Perivitelline space	[166, 171, 172]
Prostate cancer	Human	SR	Casper		Embryo (2 dpf)/juvenile	Sinus venous/subcutaneous injection	[173]
Rhabdomyosarcoma	Zebrafish	SR	AB	10–2 × 10^4^	Adult	Intraperitoneal cavity	[174]
Stomach adenocarcinoma	Human	SR	Tg(fli1:eGFP)/alb(albinos)		Embryo (2 dpf)	Yolk sac	[162]
Testicular germ cell tumor	Zebrafish	SR	AB/TU	5 × 10^3^	Adult	Intraperitoneal cavity	[175]

*SR* surgical resection, *BM* bone marrow, *PB* peripheral blood, *Pl* plasma

The availability of zebrafish transgenic lines with fluorescent labeling of blood vessels and endothelial cells, e.g. tg(fli1a:eGFP), makes the embryo stage highly suitable for detecting neoangiogenic events stimulated by cancer crosstalk processes [166, 170]. A new model recently development by Gaudenzi et al. showed the proangiogenic activity of some neuroendocrine tumors injected into 2 dpf embryos [166]. The model is based on the injection of tumor cells into the subperidermal space and on the evaluation of the growth of sprouting vessels originating from the subintestinal vein (SIV) plexus [166].

The fluorescent circulatory system of these transgenic lines and the transparency of the embryo body are also useful for assessing the metastatic potential through the detection of extravasated cells. The validation of the zebrafish system as a tool for measuring tumor invasiveness has been performed with both cell lines and primary cultures [162, 176]. This is normally done by labeling cancer cells with chemical dyes or protein stains (e.g., Red Fluorescent Protein) that emit a different fluorescent signal to that of the engineered vessels [177]. Furthermore, only a few cells need to be injected into the fish, an important advantage when there is only a small amount of primary tumor material available. 2 dpf embryos can normally tolerate grafts of 50–2000 cells without signs of toxicity, and the same results are obtainable with 50–100 cells transplanted in the blastula stage [162, 177]. Teleosts lack a number of corresponding mammalian organs with a high incidence of cancer in the human population, e.g. lung, breast and prostate. This characteristic, together with incomplete embryo development, limit the possibility of using orthotopic PDXs in this species. Over the years, several sites of injection have been tested to validate whether fish models can be used to simulate different cancer stages. The egg develops in the blastula 2.25 hpf [178] and the yolk is the only possible site of inoculation at this stage. However, the majority of studies select 2 dpf embryos as PDX recipients [72, 105, 163, 165, 178] which, unlike blastula, offer a greater number of injection possibilities. Two other inoculation sites that permit the release of cancer cells directly into the blood circulation are the duct of Cuvier (situated in the upper part of the yolk sac) and the cardinal vein (tail region) [50, 169]. Neuro-oncology studies have also reported successful orthotopic engraftments inside the hindbrain ventricles [165]. Eden et al. tested this approach in the cerebral hemisphere of juvenile fish (30 dpf) using mouse-derived glioblastoma cells [164]. Histology confirmed the preservation of mouse tissue characteristics and fluorescent imaging showed a reproducible growth rate and spinal metastases [164].

Recently, adult zebrafish have begun to be used for PDXs [165]. The possibility of monitoring tumor behavior for a prolonged period of time and of performing cross-species oncogenomic manipulations make adult fish an optimal instrument for cancer research. Unlike embryos, adult zebrafish have a fully developed immune system, making ablation processes necessary to prevent implant rejection. Irradiation and chemical treatments are two of the most common methods used to induce immunosuppression. Single radiation doses as low as 20 Gy or the administration of 25–250 µg/mL of dexamethasone are sufficient to ablate T cells [179, 180]. Recently, immunocompromised transgenic lines have been created such as rag2(E450fs) and Tg(zap70^y442^) [181, 182]. The advantages of a transparent body, typical of the embryo stage, can also be maintained in adults. In fact, the casper transgenic line lacks two of the three cell populations responsible for pigmentation. This line is a combination of two other transgenic fish, i.e. *nacre mutant* with arrested transcription of *mifta* gene involved in melanocyte development, and *roy orbison (roy)* characterized by a complete lack of iridophores and sparse melanocytes [167, 183]. The double mutant casper line (roy^−/−^; nacre^−/−^) shows a completely transparent phenotype of the adult body. Although casper zebrafish are still not used for primary culture transplantation, they remain a potentially useful option for the future.

Despite the aforementioned advantages of the zebrafish model, its use as a xenograft platform is not without problems. The relatively recent introduction of this animal system has left the market unprepared, and there are still few commercially available zebrafish antibodies or specific molecular kits. The incomplete immune system of embryos prevents the rejection of PDXs but also reduces the translational power of preclinical evaluations given that immune cells are involved in several cancer processes. For example, the efficacy of immunotherapeutic drugs, which interact with lymphoid cells, cannot be assessed. Furthermore, the zebrafish genome does not include all the human genes involved in tumor pathways, e.g. BRCA1, p16, IL6, LIF etc. [156]. Thus, future research should aim at developing transgenic fish that express the human molecules lacking in the zebrafish genome to close the biological gap between these two models.

## Conclusions

Translational preclinical research is acknowledged as a valuable investigational tool through which to improve our understanding of the cancer process. For decades, the gold standard for this kind of research was cell line experimentation. However, the length of time that cells are maintained in culture on monolayer supports and the processes used to obtain an immortalized phenotype both contribute to altering the original nature of the cell population. Closer collaboration between clinicians and researchers, together with improved laboratory methodological approaches, have led to primary cultures becoming a promising new option in the area of cancer research. This system has the advantage of maintaining the original phenotype of the lesion and of preserving the original tumor features, both essential for the reproduction of the tumor microenvironment. The complexity of the manipulation methods and the generally small quantity of biological material available make the management of primary cultures more complicated than that of cell lines. New approaches have been developed to overcome some of these disadvantages. 3D cultures and bioreactors represent innovative models for the implementation of microenvironment elements that cannot be reproduced by ex vivo samples. Each system offers potentialities in different aspects of tumor biology. Thus, the choice of model takes on an essential importance to guarantee the greatest advantages from primary cultures.

In conclusion, although primary cultures represent an excellent preclinical tool for the reproduction of cancer in in vitro systems, correct sample manipulation based on the processing method selected is essential to maintain the acknowledged advantages of the model.

## Abbreviations

ATP: adenosine triphosphate
CAF: cancer associated fibroblast
CCV: common cardinal vein
CSC: cancer stem cell
ECM: extracellular matrix
EDTA: ethylenediaminetetraacetic acid
EGF: epidermal growth factor
EGFR: epidermal growth factor receptor
EGTA: ethylene glycol-bis(β-aminoethyl ether)-*N*,*N*,*N*′,*N*′-tetraacetic acid
EMT: epithelial mesenchymal transition
EPC: endothelial progenitor cell
FBS: fetal bovine serum
FGF: fibroblast growth factor
GEMM: genetically engineered mouse model
HER2: human epidermal growth factor receptor 2
HGF: hepatocyte growth factor
ICC: intrahepatic cholangiocarcinoma
IL-6: interleukin 6
LIF: leukemia inhibitory factor
LOX: lysyl oxidase
MDM2: mouse double minute 2 homolog
MDSC: myeloid-derived suppressor cell
MMPs: matrix metalloproteinases
MSC: mesenchymal stem cell
NET: neuroendocrine tumor
NF-Kβ: nuclear factor kappa-light-chain-enhancer of activated B cells
NSCLC: non-small cell lung cancer
PAR: protease-activated receptor
PCL: poly(caprolactone)
PCR: polymerase chain reaction
PDLLA: poly(d,l-lactide)
PDX: patient-derived xenograft
PEG: polyethylene glycol
PMMA: poly(methyl-methacrylate)
PS: polystyrene
PU: polyurethane
SIV: subintestinal vein
TERT: telomerase reverse transcriptase
TGF-β: transforming growth factor-β
VEGF: vascular endothelial growth factor
